# Epidemiology and outcomes of anal abscess in patients on chronic dialysis: a 14-year retrospective study

**DOI:** 10.6061/clinics/2019/e638

**Published:** 2019-03-14

**Authors:** Meng-Hsuan Hsieh, Yueh-An Lu, George Kuo, Chao-Yu Chen, Wei-Chiao Sun, YuJr Lin, Ya-Chung Tian, Hsiang-Hao Hsu

**Affiliations:** I Department of Internal Medicine, Linkou Chang Gung Memorial Hospital, Chang Gung University, Taoyuan, Taiwan.; II Department of Nephrology, Kidney Research Center, Linkou Chang Gung Memorial Hospital, Chang Gung University, Taoyuan, Taiwan.; III Research Services Center For Health Information, Chang Gung University, Taoyuan, Taiwan.

**Keywords:** End-stage Renal Disease, Chronic Dialysis, Anal Abscess, Anal Fistula, Treatment

## Abstract

**OBJECTIVES:**

We conducted this retrospective study to elucidate the clinical presentation and outcomes of anal abscess in chronic dialysis patients.

**METHODS:**

We performed a chart review of patients who were hospitalized for anal abscess from Jan. 2002 to Dec. 2015. A total of 3,074 episodes of anal abscess were identified. Of these, 43 chronic dialysis patients with first-time anal abscess were enrolled. Patients were divided into a surgical group and a nonsurgical group according to the treatment received during hospitalization. The baseline characteristics, clinical findings, treatments and outcomes were obtained and analyzed. The endpoints of this study were in-hospital mortality, one-year mortality and one-year recurrence.

**RESULTS:**

Of the 43 patients, 27 (62.7%) received surgical treatment, and 16 (37.2%) received antibiotic treatment alone. There was no significant difference in age, sex, body mass index, smoking habits, comorbidities, or dialysis characteristics between the two groups. Perianal abscess was the most common type of anal abscess, and 39.5% of patients experienced fistula formation. Most patients had mixed aerobic and anaerobic flora. Our data demonstrate that there was no significant difference in hospital stay, one-year survival or recurrence rate between the surgical group and nonsurgical group. However, there was a trend toward better in-hospital survival in patients who received surgical treatment (*p*=0.082).

**CONCLUSION:**

In chronic dialysis patients with anal abscess, there was no statistically significant difference in clinical presentation and outcomes between the surgical and nonsurgical groups, although the surgical group had a trend of better in-hospital survival.

## INTRODUCTION

Anal abscess is one of the most common anorectal diseases that often occurs in young males between the ages of 30 and 50 years. The abscesses originate from cryptoglandular infection of proctodeal glands in the intersphincteric space. They are classified into perianal, ischiorectal, intersphincteric, and supralevator types based on the anatomical location (1). Patients present with acute onset of pain and swelling in the perianal region. The incidence is 16.1-20.2 per 100,000 per year, and the rate of subsequent fistula formation following an abscess is 15.5% (2,3). The reported risk factors of anal abscess include diabetes mellitus (DM), obesity, alcohol use, recent smoking, high daily salt intake, sedentary lifestyle, straining at defecation and psychosocial stress (3,4,5,6). Surgical incision and drainage are usually recommended even in cases of spontaneous perforation (7,8). Surgical treatment helps for decompression and pain relief of the abscess and prevents pelvic sepsis or Fournier's gangrene formation. Antibiotic treatment alone is thought to be inappropriate and leads to treatment failure, disease recurrence, and fistula formation.

Patients with end stage renal disease (ESRD) who need chronic dialysis have an increased operative risk for anorectal surgery because they have one or more comorbidities, anemia from chronic kidney disease and uremia-related platelet dysfunction. Considering the intraoperative risk and the postoperative care, treatment of anal abscess in chronic dialysis patients tends to be more conservative than in the general population. However, only a few small case series of anal abscess in chronic dialysis patients can be found in the literature (9,10). The clinical course and outcomes in dialysis patients with anal abscess have not been well described. This study was conducted to elucidate the clinical presentations, patient survival and recurrence of anal abscess in hospitalized chronic dialysis patients. The study also evaluated the outcomes in patients with and without surgical treatment during hospitalization.

## MATERIALS AND METHODS

This study was performed by retrospectively reviewing the medical records from all consecutive patients who were hospitalized for anal abscess. The study was conducted in a tertiary hospital in Taiwan (Chang Gung Memorial Hospital, Linko, Taiwan) from Jan. 2002 to Dec. 2015. Patients with a discharge diagnosis of anal abscess, which was defined as International Classification of Diseases, Ninth Revision, Clinical Modification (ICD-9-CM) code 566, were identified. Chronic dialysis patients were identified by using chart review and the charge codes of hemodialysis or peritoneal dialysis. The date of admission was defined as the index date.

**Figure 1:**
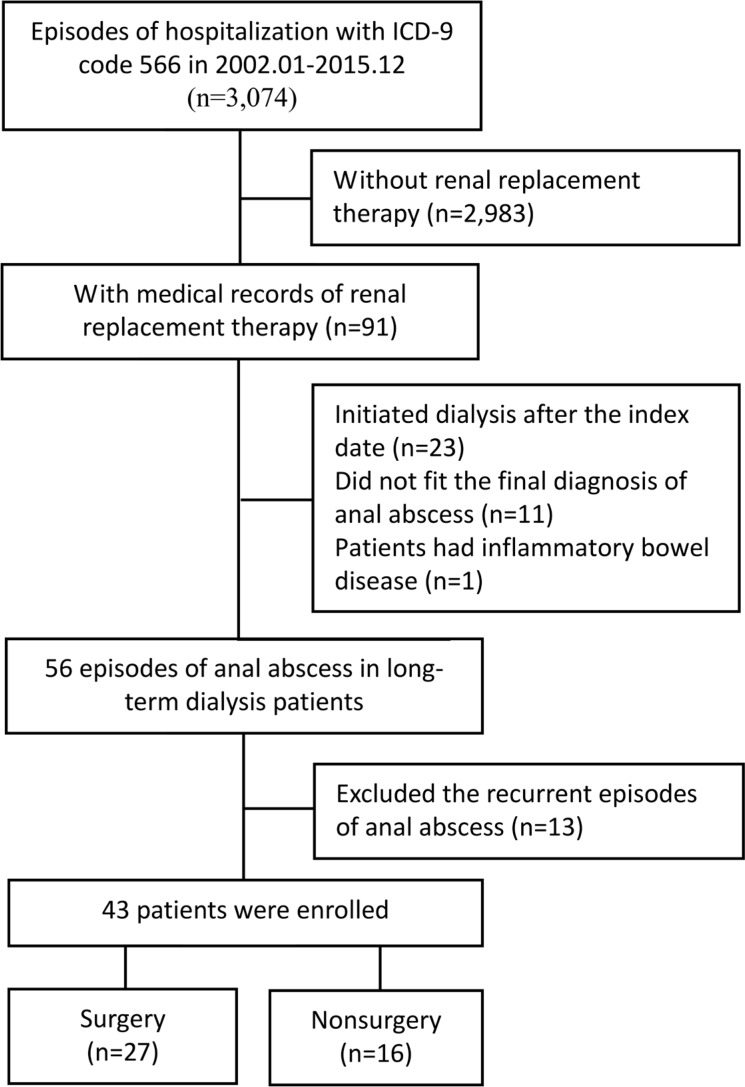
Flow chart of patient selection.

A total of 3,074 episodes of anal abscess were found in the 14-year study period using the ICD-9-CM code 566. Among these, 91 had incident dialysis. All dialysis patients were above 20 years of age. We excluded patients who initiated dialysis after the index date (n=23), patients who did not fit the diagnosis of anal abscess in chart review (n=11) and patients who had inflammatory bowel disease (n=1). Recurrent episodes of anal abscess were also excluded (n=13). Forty-three chronic dialysis patients who were hospitalized for first-time anal abscess were enrolled and analyzed (Figure 1).

The study reviewed the patient's baseline characteristics and comorbidities. The dialysis-associated factors, including primary cause of ESRD, duration of renal replacement therapy and type of dialysis access, were also documented. Patients were divided into two groups according to whether they received surgical intervention during the index admission. The surgical procedure was defined as any incision and drainage of the abscess with or without anal fistulotomy and fistulectomy. We also recorded the clinical presentation, the type of abscess, the microbiology and the occurrence of fistula formation. Patient outcomes were evaluated during hospitalization and 1 year after admission. The endpoints of this study were in-hospital mortality, one-year mortality and one-year recurrence.

Analysis was conducted using R 3.2.4 software (R Project for Statistical Computing, Vienna, Austria). Descriptive statistics are presented as the mean ± standard deviation for continuous data and count (%) for categorical data. The t-test and Fischer's exact test were applied in the comparison of surgically and nonsurgically treated patients. A two-sided *p*-value of 0.05 was considered statistically significant. The follow-up time ended when patients died, relapsed, or were recurrence-free for one year.

### Ethics

The study methods are in accordance with the ethical standards of the responsible committee on human experimentation and with the Helsinki Declaration. The Institutional Review Board of Chang Gung Memorial Hospital approved this study (IRB Approval No. 2016009714B0).

## RESULTS

Forty-three dialysis patients who were hospitalized for anal abscess for the first time were identified in 3,074 episodes of anal abscess in the 14-year study period. The baseline characteristics of the chronic dialysis patients with anal abscess are illustrated in Table 1. The average age was 59.98±11.93 years old. Male predominance was noted in these patients (n=33, 76.7%). The body mass index (BMI) was 24.20±3.89 kg/m^2^; 37.2% of the patients (n=16) had recently smoked; 53.5% of the patients had hypertension (n=25); 58.1% of the patients had DM (n=25); 14.0% of the patients had coronary artery disease (n=6); 7.0% of the patients had congestive heart failure (n=3); 16.3% of the patients had a cerebral vascular accident (n=7); 9.3% of the patients had chronic hepatitis B (n=4); 25.6% of the patients had chronic hepatitis C (n=11); 16.3% of the patients had liver cirrhosis (n=7); and 16.3% of the patients had malignancy (n=7). We also found that 62.8% of the patients had constipation (n=27), and 16.3% of the patients had hemorrhoids (n=7). The two major primary causes of ESRD were diabetic nephropathy (n=15, 34.9%) and chronic glomerulonephritis (n=23, 53.5%). The duration of renal replacement therapy ranged from 2 months to 28 years (median=3.94 years). In these patients, 65.1% used an arteriovenous fistula (n=28), 14.0% used an arteriovenous graft (n=6), 7% used a tunneled cuffed catheter (n=3), 2.3% used a nontunneled central venous catheter (n=1), and 11.6% used peritoneal dialysis (n=5) as their dialysis accesses. The study showed that 62.8% of chronic dialysis patients with anal abscess (n=27) received surgical treatment during hospitalization, and 37.2% of patients (n=16) received medical treatment alone. There was no significant difference in age, sex, BMI, recent smoking, comorbidities, or dialysis characteristics between the surgical and nonsurgical groups (Table 1).

**Table 1:** Baseline characteristics of chronic dialysis patients with anal abscess.

Characteristics		All (N=43)	Surgical (N=27)	Nonsurgical (N=16)	*p*-value
Age, year		59.98±11.93	59.59±12.35	60.63±11.56	0.788
Male gender, n (%)		33 (76.7)	21 (77.7)	12 (75.0)	1.000*
Body mass index, kg/m^2^		24.20±3.89	24.12±4.06	24.38±3.62	0.857
Recent smoking, n (%)		16 (37.2)	11 (40.7)	5 (31.3)	0.534
Comorbidity, n (%)					
	Hypertension	23 (53.5)	14 (51.9)	9 (56.2)	0.780
	Diabetes mellitus	25 (58.1)	17 (63.0)	8 (50.0)	0.405
	Coronary artery disease	6 (14.0)	2 (7.4)	4 (25.0)	0.174*
	Congestive heart failure	3 (7.0)	2 (7.4)	1 (6.3)	0.545*
	Cerebral vascular accident	7 (16.3)	3 (11.1)	4 (25.0)	0.394*
	Chronic hepatitis B	4 (9.3)	1 (3.7)	3 (18.8)	
	Chronic hepatitis C	11 (25.6)	6 (22.2)	5 (31.3)	
	Cirrhosis	7 (16.3)	2 (7.4)	5 (31.3)	0.082*
	Malignancy	7 (16.3)	3 (11.1)	4 (25.0)	0.394*
	Constipation	27 (62.8)	19 (70.4)	8 (50.0)	0.182
	Hemorrhoids	7 (16.3)	5 (18.5)	2 (12.5)	0.695*
Primary cause of end-stage renal disease					1.000*
	Diabetic nephropathy	15 (34.9)	9 (33.3)	6 (37.5)	
	Chronic glomerulonephritis	23 (53.5)	15 (55.5)	8 (50.0)	
	Others	5 (11.6)	3 (11.1)	2 (12.5)	
Duration of renal replacement therapy, median days, (lower quartile-upper quartile)		1,439 (633-2,779)	1,381 (678-1,813.5)	2,001.5 (730.25-3,091.5)	0.462#
Type of dialysis access, n (%)					0.769*
	Arteriovenous fistula	28 (65.1)	17 (63.0)	11 (68.8)	
	Arteriovenous graft	6 (14.0)	4 (14.8)	2 (12.5)	
	Tunneled cuffed catheter	3 (7.0)	1 (3.7)	2 (12.5)	
	Nontunneled central venous catheter	1 (2.3)	1 (3.7)	0 (0.0)	
	Peritoneal dialysis	5 (11.6)	4 (14.8)	1 (6.3)	

* Fisher's exact test.

# Mann-Whitney test.

Less than half of the patients presented with fever (n=21, 48.8%) at admission. Perianal abscess (n=37, 86.0%) was the most common type of abscess in both the surgical and nonsurgical groups, whereas ischiorectal abscess (n=3, 7.0%), intersphincteric abscess (n=2, 4.7%) and supralevator abscess (n=1, 2.3%) were also found in chronic dialysis patients. Seventeen patients (39.5%) had an anal fistula the first time they were diagnosed with anal abscess. There was no significant difference in clinical presentation or type of abscess between the surgical and nonsurgical groups (Table 2).

**Table 2:** Clinical characteristics and outcomes of chronic dialysis patients with surgical and nonsurgical treatments.

Characteristics		All (N=43)	Surgical (N=27)	Nonsurgical (N=16)	*p*-value
Fever, n (%)		21 (48.8)	13 (48.1)	8 (50.0)	0.907
Type of abscess, n (%)					
	Perianal	37 (86.0)	22 (81.5)	15 (93.8)	0.386*
	Ischiorectal	3 (7.0)	3 (11.1)	0 (0.0)	0.282*
	Intersphincteric	2 (4.7)	1 (3.7)	1 (6.3)	1.000*
	Supralevator	1 (2.3)	1 (3.7)	0 (0.0)	1.000*
Fistula formation, n (%)		17 (39.5)	10 (37.0)	7 (43.8)	0.663
Microbiology, n (%)					0.077*
	Mixed aerobic and anaerobic flora	16 (37.2)	13 (48.1)	3 (18.8)	
	Gram-positive and Gram-negative aerobic bacteria	3 (7.0)	1 (3.7)	2 (12.5)	
	Gram-positive aerobic bacteria only	6 (14.0)	4 (14.8)	2 (12.5)	
	Gram-negative aerobic bacteria only	3 (7.0)	3 (11.1)	0 (0.0)	
	Anaerobic bacteria only	1 (2.3)	0 (0.0)	1 (6.3)	
	No culture result	14 (32.6)	6 (22.2)	8 (50.0)	
Laboratory examination					
	White blood cell count, cells/ml	14,519.05±7,204.28	13,253.33±4,520.09	15,222.22±8,331.00	0.403
	Hemoglobin, g/dL	10.21±1.83	9.91±1.91	10.38±1.80	0.427
	C-reactive protein, mg/mL	158.56±100.29	172.38±97.70	152.74±103.43	0.651
	Albumin, g/dL	3.07±0.53	3.20±0.82	3.00±0.35	0.626
Antibiotic treatment		40 (93.0)	25 (92.6)	15 (93.8)	1.000*
Hospital stay, days		20.70±21.11	19.33±23.87	23.00±15.87	0.588
In-hospital survival, n (%)		36 (83.7)	25 (92.6)	11 (68.8)	0.082*
One-year survival, n (%)		33 (76.7)	23 (85.2)	10 (62.5)	0.137*
One-year recurrence, n (%)		5 (13.9)	3 (12.0)	2 (18.2)	0.631*

* Fisher's exact test.

The most common pathogens grown in abscess cultures were mixed aerobic and anaerobic flora (n=16, 37.2%). Three patients had both Gram-positive aerobic and Gram-negative aerobic bacteria (7.0%), 6 patients had Gram-positive aerobic bacteria alone (14.0%), 3 patients had Gram-negative aerobic bacteria alone (7.0%), and 1 patient had anaerobic bacteria (2.3%). Most patients who did not have a culture result (n=14, 32.6%) did not send an abscess culture. The isolated microorganisms from anal abscesses are shown in Table 3. *Streptococcus* spp., *Enterococcus* spp., and *Staphylococcus* spp. were common Gram-positive aerobic bacteria. *Escherichia coli* and *Klebsiella* spp. were common Gram-negative aerobic bacteria. *Bacteroides* spp. was the most common anaerobic bacteria in this study. Yeast was also noted in 13.8% of the positive cultures.

**Table 3:** Isolated microorganisms from anal abscesses of chronic dialysis patients.

Microorganisms		n (%)
Gram-positive aerobic bacteria		
	*Streptococcus* spp.	8 (27.6)
	*Enterococcus* spp.	6 (20.7)
	*Staphylococcus aureus*	4 (13.8)
	Coagulase-negative *Staphylococci*	2 (6.9)
	*Stenotrophomonas maltophilia*	1 (3.4)
Gram-negative aerobic bacteria		
	*Escherichia coli*	10 (34.5)
	*Klebsiella* spp.	4 (13.8)
	*Proteus* spp.	2 (6.9)
	*Citrobacter diversus*	1 (3.4)
	*Acinetobacter junii*	1 (3.4)
*Anaerobic bacteria*		
	*Bacteroides* spp.	14 (48.3)
	*Prevotella* spp.	4 (13.8)
	*Peptostreptococcus* spp.	3 (10.3)
	*Fusobacterium* spp.	3 (10.3)
	*Clostridium* spp.	1 (3.4)
	*Veillonella* spp.	1 (3.4)
Yeast		4 (13.8)

Chronic dialysis patients with anal abscess often develop leukocytosis, anemia, elevated C-reactive protein and hypoalbuminemia. Among the 43 cases, 93.0% received intravenous or oral antibiotic treatment (n=40). Physicians often used two combined antibiotics empirically, such as ceftriaxone plus metronidazole, to cover the mixed aerobic and anaerobic flora. Antibiotics were adjusted according to the culture results. Two patients in the surgical group and one patient in the nonsurgical group did not receive intravenous or oral antibiotics. They received local antibiotic ointment for wound care. In the patients who underwent surgical intervention, 44.4% received local anesthesia (n=12), 25.9% received regional anesthesia (n=7), and 29.6% received general anesthesia (n=8).

The patient's hospital stay was 20.70±21.11 days (median=16, ranged from 1-78). The in-hospital survival rate was 83.7% (n=36), the one-year survival rate was 76.7% (n=33), and the one-year recurrence rate of patients who survived during hospitalization was 13.9% (n=5). Septic shock and respiratory failure were causes of death in all patients who died during hospitalization. Patients died from septic shock due to anal abscess or an anal abscess accompanied by pneumonia, spontaneous bacterial peritonitis or blood stream infection. The causes of death of patients who died after discharge were sepsis and sudden death that was not related to a recurrence of anal abscess. There was no significant difference in survival rate or recurrence rate between the surgical and nonsurgical groups. However, there was a trend toward a better in-hospital survival rate in patients who underwent surgery for anal abscess at admission (92.6% *vs*. 68.8%, *p*=0.082).

## DISCUSSION

This study reviewed 43 dialysis patients with first-time anal abscess from a tertiary medical center within a 14-year study period. The male to female ratio was 3.29 in dialysis patients with anal abscess. The average age in the study cohort was older than that of nondialysis patients (11). Chronic constipation and DM, which are commonly noted in chronic dialysis patients, are well-established risk factors of anal abscess. The study also revealed a high prevalence of chronic hepatitis (9.3% of hepatitis B and 25.6% of hepatitis C) and cirrhosis (16.3%) in chronic dialysis patients. A case series reported that two of eight patients with Fournier's gangrene had liver cirrhosis or hepatoma (12). Although the association between liver disease and perianal infection had not been described in a large population-based study in Europe (3), further study in the chronic dialysis populations may be considered.

Anal abscess may impair patient's quality of life because of the resulting pain, discomfort, anxiety and depression. Although surgical incision and drainage have been the standard treatment, 37.2% of the chronic dialysis patients received conservative treatment alone. Most of the patients in our study received a proctologist consultation during hospitalization. Sheikh et al. suggested that anorectal surgery was well tolerated in chronic hemodialysis patients on a well-managed dialysis program (10). For the patients who did not receive surgical treatment, surgery was not recommended by proctologists because of an unstable clinical condition, spontaneous perforation of the abscess, liquification of the abscess or excellent response to antibiotic treatment. The study revealed no significant difference in patient outcomes between the surgical and nonsurgical groups, whereas there was a trend toward a better in-hospital survival rate in patients who received surgery at admission. Although surgical intervention might provide a survival benefit, it is possible that patients receiving conservative treatment had more severe clinical complications that resulted in poorer outcomes.

Chronic dialysis patients with anal abscess had a high incidence of fistula formation (39.5%). Additionally, more than 90% of the patients received antibiotic treatment. In a prospective study of members of the general population with primary anal abscess in the United Kingdom, only 14.6% of the patients had an anal fistula when they were diagnosed with anal abscess, and 18.3% of the patients received postoperative antibiotics (13). Other studies revealed that 22.3-27.1% of the adult patients had an anal fistula (14,15). Fistula results from chronic inflammation of the perianal area that connects the abscess to skin. The reported predictors of fistula formation include inflammatory bowel disease, female sex, age and intersphincteric or ischiorectal abscess (2). A previous meta-analysis showed that treatment of an anal fistula at the same time as drainage of perianal abscess reduces the chances of recurrent abscess and repeat surgery (16). A recent randomized trial showed that postoperative prophylactic antibiotic therapy targeting mixed aerobic and anaerobic pathogens protected patients from fistula formation (14).

Most of the pathogens in anal abscess come from the gastrointestinal tract and skin. Abscess cultures have often showed mixed aerobic and anaerobic flora. *Escherichia coli*, *Staphylococci* spp. and *Klebsiella pneumoniae* were common aerobic pathogens and *Bacteroides* spp. and *Peptostreptococcus* spp. were common anaerobic pathogens in nondialysis patients (15,17). The culture results of chronic dialysis patients were similar to those of nondialysis patients. Current evidence suggests that patients with ESRD are characterized by impaired native and adaptive immunity (18,19). Although postoperative antibiotics had a limited role in uncomplicated anal abscess (1,20), antibiotic therapy may be considered in all chronic dialysis patients because of their immunosuppressive *status*. The choice of empiric antibiotics should adequately cover both aerobic and anaerobic flora.

This study was limited by the small number of cases, the single-center experience and its retrospective design. Only 43 dialysis patients were identified in 14 years. There may have been selection bias imposed by the inclusion of a tertiary center. Despite these limitations, to our knowledge, this is the first study to focus on chronic dialysis patients with anal abscesses and report the outcomes in surgical and nonsurgical groups.
